# Experimental Study on Shear Wave Transmission in Fractured Media

**DOI:** 10.3390/s22114047

**Published:** 2022-05-26

**Authors:** Ming Cai, Hongliang Wu, Yi Xin, Peng Liu, Chengguang Zhang, Jun Tang, Minjie Lin, Lihong Tan

**Affiliations:** 1 Key Laboratory of Exploration Technologies for Oil and Gas Resources, Ministry of Education, Yangtze University, Wuhan 430100, China; caiming@yangtzeu.edu.cn (M.C.); tangjun@yangtzeu.edu.cn (J.T.); tanlihong1998@163.com (L.T.); 2 College of Geophysics and Petroleum Resources, Yangtze University, Wuhan 430100, China; 3 PetroChina Research Institute of Petroleum Exploration &Development, Beijing 100083, China; wuhongliang@petrochina.com.cn (H.W.); liupeng1987@petrochina.com.cn (P.L.); linmj@petrochina.com.cn (M.L.); 4 Exploration and Development Research Institute of Tarim Oilfield Company, PetroChina, Korla 841000, China; xinyi-tlm@petrochina.com.cn

**Keywords:** shear wave signal, fracture, extraction of wave attenuation, array acoustic logging, sonic experiment, sonic transducer

## Abstract

Unconventional oil and gas reservoirs have broad exploration and development prospects. Fracture parameters and effectiveness evaluation are two of the key tasks for the evaluation of these types of reservoirs. Array acoustic logging can be used for fracture evaluation to compensate for the deficiencies of the image logging fracture evaluation method. Therefore, to develop acoustic logging evaluation methods as well as nondestructive testing methods for fractures, experiments were conducted to study the shear wave transmission in fractured media. Experiment data demonstrate a good correlation between the shear wave attenuation coefficient and fracture width, and the shear wave attenuation coefficients rise logarithmically with the increase in the fracture width for all models with different porosities and distinct dip angles of fractures. The shear wave attenuation coefficient changes relatively faster with the fracture width when the fracture width is within 250 μm. In addition, the shear wave attenuation is affected by the core porosity and fracture dip angle. When the fracture width is constant, the shear wave attenuation caused by the 0° fracture is relatively larger and is obviously greater than that of the fractures at other angles, which is consistent with the existing experimental results. The results of this study can be used to guide further research on amplitude compensation methods for sonic signal transmission in fractured media and fracture evaluation methods.

## 1. Introduction

The oil and gas stored in the pores and fractures of underground formations are extremely important resources for human survival and social development. With the development of oil and gas exploration, tight oil and gas reservoirs play increasingly important roles in stabilizing and increasing production throughout the world, especially in North America and China [1,2]. Tight oil and gas reservoirs have been defined as any reservoir with porosity less than 10% and permeability less than 0.1 mD, or a reservoir that will not produce commercially without stimulation [2]. In China, the number and proportion of fractured tight reservoirs are more prominent. More than half of the total production of oil and gas in China is ensured by tight reservoirs, as well as oil and gas reserves, which account for more than two-thirds of the total reserves to be put into production in the future [3]. Thus, tight reservoirs, especially fractured tight reservoirs, have broad exploration and development prospects [2]. In recent years, with the exploration and development of fractured tight reservoirs, such as shale gas reservoirs, fractured reservoirs, and deep fractured tight sandstone reservoirs, the evaluation methods of these reservoirs have become a hot research field [4,5,6]. For these types of reservoirs, fractures provide pathways for fluid flow and storage spaces for tight oil and gas, and fracture development is the main reason for their high production [6,7]. Therefore, fracture identification and characteristic parameter evaluation are very important evaluation tasks for these types of reservoirs.

Geophysical well-logging data can be used to identify and quantitatively evaluate fractures, and it is the most important fracture evaluation method. Scholars all over the world have conducted a lot of research on geophysical fracture evaluation methods, which mainly include conventional logging (lateral logging or induction logging, acoustic velocity logging, and density logging) fracture evaluation method [8,9], image logging (micro-resistivity imaging and ultrasonic imaging) fracture evaluation method [5,10,11,12], array acoustic logging fracture evaluation method [6,13,14,15,16], and acoustic reflection imaging fracture evaluation method [17,18,19,20]. The conventional logging evaluation method is generally used to qualitatively assess fractures. The image logging (e.g., FMI) evaluation method is presently considered to be the most reliable logging fracture evaluation technology. It can estimate quantitative fracture parameters, such as fracture aperture, dip angle, length, density, and porosity. However, the application effect of the micro-resistivity imaging evaluation method is of poor quality in oil-base mud wells, and the application effect of the ultrasonic imaging evaluation method is easily affected by irregular borehole and mud particle scattering. Moreover, the investigation depth of the micro-resistivity imaging and the ultrasonic imaging are both so shallow that image logging can only identify fractures located on the borehole wall and cannot estimate the lateral extent of the fractures. Thus, image logging has some shortcomings in the evaluation of fracture effectiveness, which is affected by fracture width, filling condition, and extension length. The acoustic waves in the borehole propagate within the formation around the borehole in the array acoustic logging, and fractures in the formation have a significant influence on the propagation of sonic waves. The acoustic waves in the borehole with larger investigation scopes are, therefore, used to estimate the fracture aperture, density, and lateral extent, which can make up for the shortcomings of the image logging fracture evaluation method [6,16,21,22,23]. However, the array acoustic logging fracture evaluation method is presently incomplete. Generally, the Stoneley wave is only used to identify fractures and evaluate formation permeability, while the dipole shear wave is just used to evaluate fracture anisotropy parameters [14]. The acoustic reflection imaging with the largest investigation scope can detect reflectors that are 10 m or even farther away from the borehole [17,19,24], and it is, therefore, more suitable for the evaluation of fracture lateral extent. However, the acoustic reflection imaging fracture evaluation method still encounters many difficulties. In particular, the response of the acoustic reflection imaging to micro-fractures needs to be studied further.

The resolutions and penetration depths of P and S waves from array acoustic logging are between that of image logging and acoustic reflection imaging [14,17,20]. Therefore, array acoustic logging is more suitable for providing information about the mesoscale fracture. In order to make full use of the information of the acoustic waves in the borehole to quantitatively evaluate fracture parameters and effectiveness, it is necessary to study the influence of fractures, especially oblique fractures, on the propagation of the acoustic waves in the borehole. Hudson theoretically studied the wave velocity and attenuation of elastic waves traveling through fractured media with randomly distributed thin, circular fractures [25]. Ass’ad et al. analyzed the relationship between wave velocity anisotropy and fracture density through the physical experiment method [26]. Wei investigated the influence of fracture density and morphology on seismic waves by using the artificial fracture model. The results of this research show that the degree of shear wave splitting is affected by fracture density [27]. In addition, the slow shear wave velocity decreases with the increase in fracture density, while the fast shear wave velocity is not affected. Finally, the effect of fracture morphology on velocity is less than that on attenuation. For their part, Lambert et al. examined the attenuation and dispersion of P-waves traveling through porous rock with flat fracture by using numerical simulation [28]. Zhu et al. researched the attenuation anisotropy of P-waves traveling through TI (Transverse Isotropy) media by utilizing physical simulation experiments [29]. Li et al. inspected the relationship between fracture density, hole density, size, shape, and P-wave properties through ultrasonic experiments [30]. The results demonstrate that P-wave property parameters are most sensitive to the densities of the hole and fracture, and, in particular, the P-wave amplitude is more sensitive than the velocity. Tillotson et al. confirmed the relationship between fracture density and S-wave splitting by using physical experiments of artificial sandstone samples with fractures [31]. Chen et al. studied the influence of fracture porosity, fracture dip angle, fracture density, and mm-level fracture width on the attenuation of ultrasonic P-waves by employing numerical simulation [32]. Bakku et al. analyzed the influence of fracture compliance on the attenuation of Stoneley waves, and proposed a method to evaluate fracture compliance and aperture by using Stoneley-wave attenuation [21,22]. Li et al. investigated the influence of the fracture number, fracture dip angle, and fracture spacing on the elastic wave velocity and attenuation in VTI (Vertical Transverse Isotropy) media by applying numerical simulation [33]. Yuan et al. worked on the relationship between flat fracture width and acoustic velocity through a core acoustic experiment [34]. The results show that the P- and S-wave velocities decrease linearly with the increase in the fracture width, with the change of P-wave velocity being more dramatic. Wei et al. conducted a physical experimental study on artificial cores with different penny-shaped fracture densities at frequencies of 0.1, 0.25, and 0.5 MHz, principally measuring the P- and S-wave velocities and anisotropies [35]. Cai et al. studied the influence of flat fracture on S-wave attenuation through physical experiments of fractured sand cores [16]. The results demonstrate that there is a good correlation between the equivalent fracture width and the S-wave attenuation coefficient; nevertheless, the influences of fracture dip angle and fracture filling conditions on experimental results were not considered. Markov et al. theoretically studied and analyzed the reflection and transmission law of P- and S-waves at the crack in a porous-fractured medium [36,37]. Pan et al. took advantage of the ratio of shear-to-compressional wave velocity to identify fluid and detect fracture [38]. Li et al. explored the identification method of gas-bearing carbonate reservoirs based on joint acoustic-resistivity experiments, and the P-wave velocity was used to identify a gas reservoir [39]. Lee et al. and Li et al. qualitatively analyzed the influence of fractures on sonic wave attenuation, and the attenuation information was applied to identify fractures. However, the quantitative parameters of fractures cannot be provided [40,41].

The studies mentioned above mainly focused on the effect of fractures on the S-wave splitting and the velocity and amplitude of the P-wave. However, a large number of experimental and field data analysis results show that shear wave attenuation is more sensitive to the change of fracture attributes [16,40,41]. The existing results qualitatively analyzed the influence of fractures on shear wave attenuation, but the quantitative experimental research on the influence of fractures, especially micro-fractures, with different dip angles and widths on shear wave attenuation has not been reported. In order to realize the fine evaluation of fracture attribute parameters by using shear wave information, the study of the influences of different fractures on the attenuation of ultrasonic S-wave with a frequency band of 20–150 kHz was conducted by using physical experiments on 12 tight sand cores with micron-level oblique fractures in this paper. Our main objective is to investigate the shear wave transmission law in fractured media and establish the quantitative relationship chart of shear wave attenuation with fracture attribute parameters. The important distinctions of our work from the previous efforts are reflected in the following two aspects: on the one hand, we established an acoustic experimental system and scheme for the cylindrical plug cores with different oblique micro-fractures, and confirmed their feasibilities; on the other hand, we quantitatively investigated the influences of oblique fracture property changes on the shear wave attenuation through acoustic physical experiments. In the following sections, the experimental principle is first described. Then, the experiment devices and measurement methods are demonstrated. Next, the shear wave measurement experiments are conducted on cores with oblique fractures, and the waveform data are processed and analyzed where the quantitative relationships between the ultrasonic shear wave attenuation and the micron-level fracture aperture, and the dip angle are mainly researched. Finally, the paper is summarized, with conclusions drawn according to the results obtained from this study.

## 2. Experimental Principle

**Figure 1:**
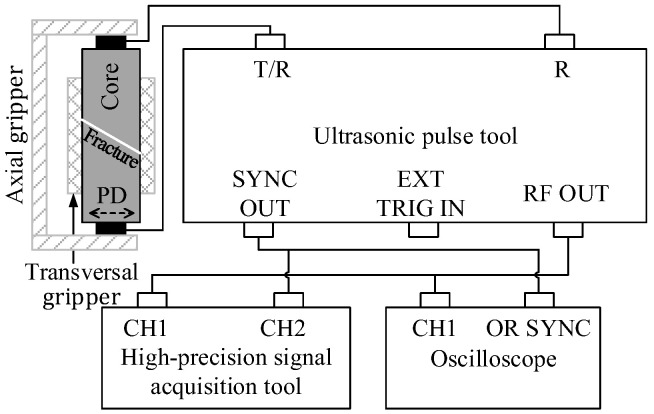
Schematic diagram of experimental measurement of core with oblique fracture. PD is the abbreviation of polarization direction of the shear wave.

A transmission method [16,34] was used to measure the ultrasonic shear wave along the longitudinal axis of the cylindrical plug core sample as shown in Figure 1, in which the dotted double arrow indicates the polarization direction (PD) of the shear wave. The standard cylindrical core sample was cut into two equal sections at a specified angle in the transverse direction to simulate fractures. In the experiment, the fracture width was adjusted and controlled by placing annular PET (Polyester) films with different thicknesses between the fracture surfaces, and the shear waveforms passing through the core samples with different fractures, which can be measured.

The shear waveforms were, respectively, measured and recorded when the two sections of the core were directly connected and different thicknesses of annular PET films were placed between the fracture surfaces with air filling the micro-gap. The shear waveform energy of the core with a fracture width of 0 μm was chosen as a reference value, and other shear waveform energies of the cores with fractures of different widths were analyzed. Subsequently, the corresponding S-wave attenuation coefficients can be calculated by the following Equation:

(1) $$\left\{ \begin{array}{l} \alpha =-20\mathrm{lg}(\frac{E}{E_{0}})\\ E=\overset{W\_end}{\underset{i=W\_start}{\sum }}A_{i}^{2} \end{array}\right.,$$

where α is the S-wave attenuation coefficient, E and $E_{0}$ are the shear waveform energies in the target time windows when the fracture width is not 0 μm and the fracture width is 0 μm, respectively, W_start and W_end are the first and last waveform sample indexes of the target time window, and $A_{i}$ is the *i*th amplitude sample value of the waveform.

Based on the above principle, shear waveform measurement experiments can be conducted on the cores with oblique fractures of different widths, then the influence of oblique micro-fractures on S-wave attenuation can be studied by processing and analyzing the waveform data.

## 3. Experimental Devices and Methods

### 3.1. Experimental Devices

The experimental system mainly included tight sand core samples; broadband piezoceramic S-wave transmitting and receiving transducers with a dominant frequency of 0.1 MHz; an ultrasonic pulse tool, which has ultrasonic pulse with a frequency range of 0.02–10 MHz generating and receiving functions; a high-precision signal acquisition tool; a digital oscilloscope; a core sample horizontal clamping device; an axial core gripper with a quantitatively adjustable axial clamping pressure; a transversal core gripper; PET films of different thicknesses; BNC (Bayonet Nut Connector) signal transmission lines; a spiral micrometer with an accuracy of 0.001 mm; and an electronic vernier caliper with an accuracy of 0.02 mm. In order to achieve a high signal-to-noise ratio (SNR) shear waveform measurement, high viscosity butter was used as the coupling material between the transducer and the core, and the waveforms were stacked 32 times [42].

### 3.2. The Construction of Physical Core Models with Oblique Fractures

**Figure 2:**
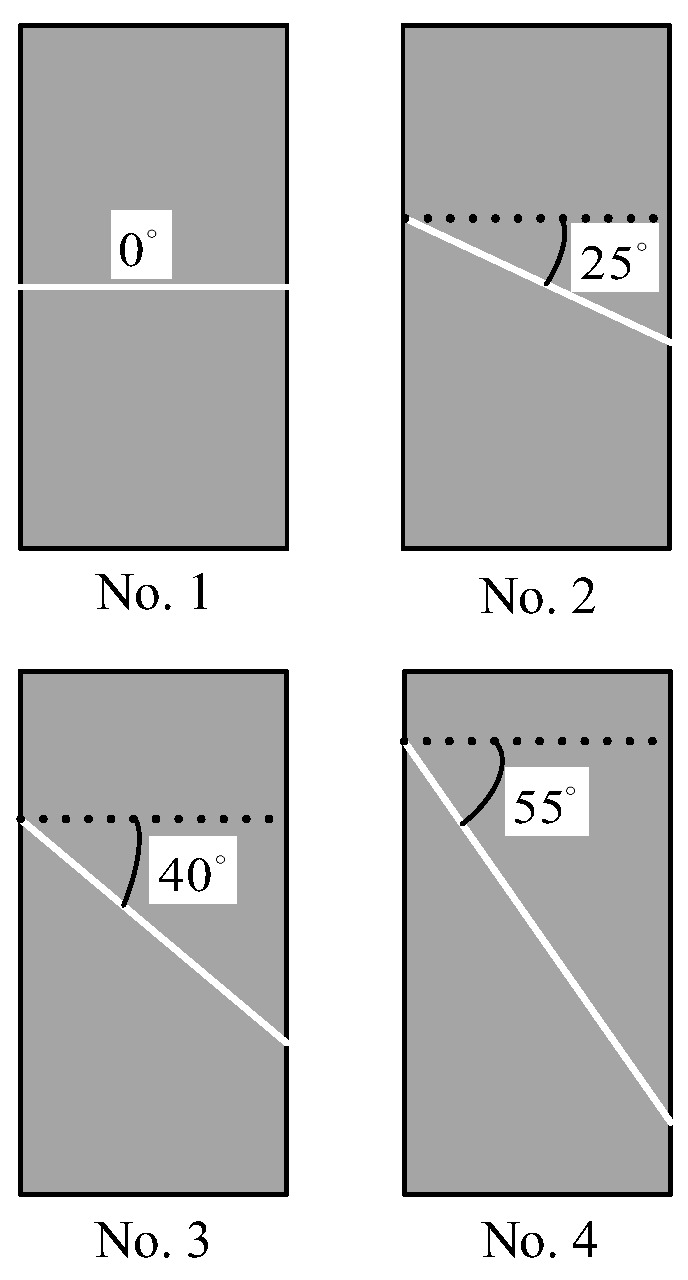
Cutting scheme of the cylindrical plug core models with oblique fractures; the white, solid line demonstrates the fracture pattern.

Based on the above experimental principle, twelve tight sand core models were built. To build core models containing fractures with different dip angles, each complete standard cylindrical core sample was cut into two halves that were approximately the same at a specified angle in the transverse direction. Subsequently, the two core halves were joined together along the cutting surface to form a core model sample containing a fracture with a specified dip angle. To ensure the cutting surfaces were flat and smooth, so that the fracture surfaces fit together well when the two core halves were joined together, the wire cutting method was preferred as a means of cutting the core. The schematic diagram of the cutting scheme of the cylindrical plug core models with fractures at different dip angles is shown in Figure 2, where the white, solid line demonstrates the fracture pattern.

**Figure 3:**
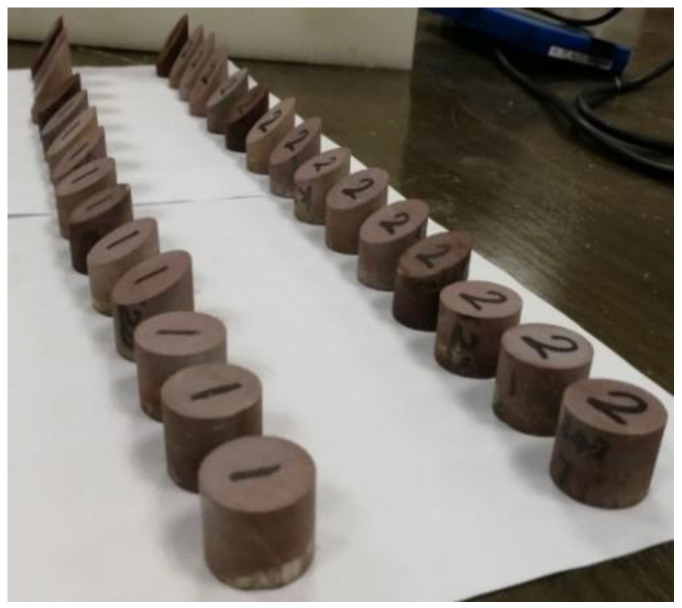
Photograph of the cut cylindrical plug core models with oblique fractures.

All the core samples in this experiment originated from the tight sandstone reservoir in the Kuqa area of the Tarim oilfield. To reduce the influence of porosity on the experimental results, three sets of core samples were selected and each set consisted of four core samples with similar porosities. The basic physical parameters of the experimental cores are listed in Table 1. Groupings of four cores with porosities between 4% and 5%, 5% and 6%, and 6% and 8% were, respectively, marked as the sets A, B, and C. Figure 3 presents the photograph of the cut cylindrical plug core models with fractures at different dip angles. The cut core samples can be used in acoustic experiments after drying, vacuuming, and pressurized water saturation.

### 3.3. Fine Adjustment Method of Fracture Parameters

In the experimental process, the fine adjustment of fracture attribute parameters was very important to improve the reliability of experimental results. There were many attribute parameters of micro-fractures. However, our experiments mainly focused on the influence of the change of the fracture dip angle and width on the S-wave attenuation. The fracture dip angle can be finely controlled throughout the cutting of the core samples. During the experiment, the fracture dip angle can be adjusted easily by selecting a core sample containing a fracture with a specified dip angle.

**Figure 4:**
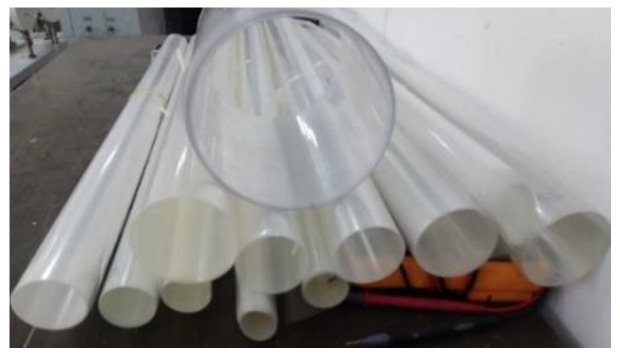
PET films with different thicknesses.

**Figure 5:**
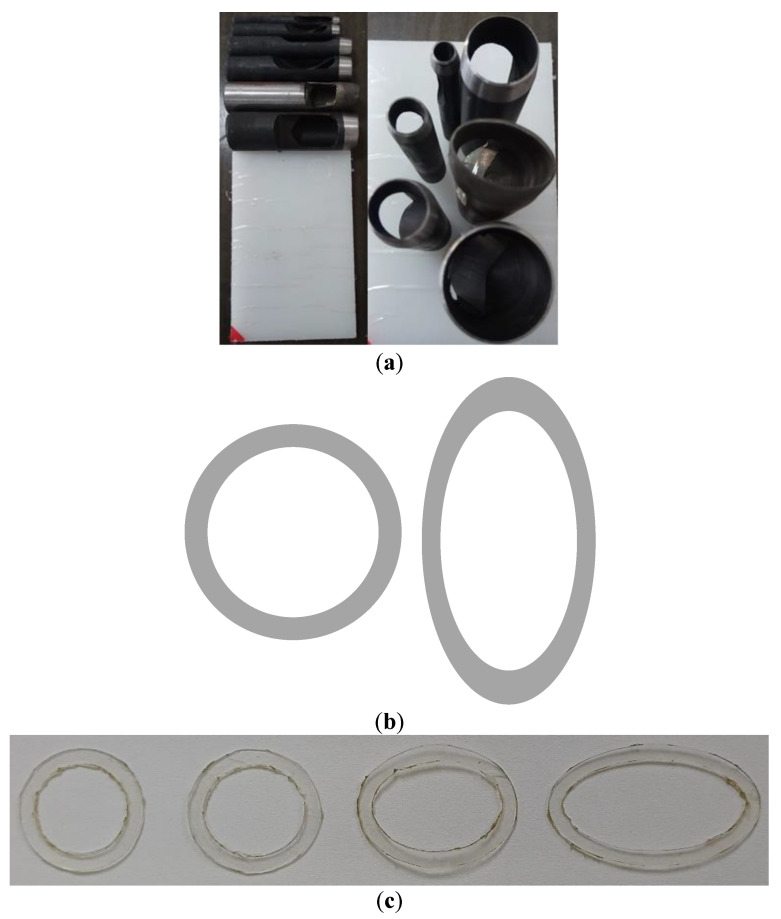
PET film gaskets with different sizes and punchers. (**a**) Photograph of punchers; (**b**) schematic diagram of gaskets; (**c**) photograph of gaskets.

To realize the fine adjustment of the fracture width, a special PET film was selected as the gasket material. This PET film can withstand high temperature and high pressure, and the test results show that the PET film does not deform for 30 min at a pressure of 40 MPa and a temperature of 100 °C. There were 12.5 μm, 25 μm, 50 μm,75 μm, 80 μm, 100 μm, 125 μm, 150 μm, 175 μm, 200 μm, 250 μm, 300 μm, 350 μm, and 500 μm thickness specifications of the PET film products in the market (as shown in Figure 4). Other thickness specifications can also be customized. For the experiment, different specifications of annular PET film gaskets can be made as needed (as presented in Figure 5). The gaskets were placed between the two core cutting surfaces and a certain clamping pressure was applied in the axial and transversal directions, respectively. As a result, the fine control of the fracture width was realized.

### 3.4. Clamping Method of a Core with an Oblique Micro-Fracture

In the acoustic measurement experiment, the surface contact between the core sample and acoustic transducer should be well established to obtain stable and high SNR waveform signals. Therefore, a uniaxial core gripper was often used to clamp the core and the transducer, and a certain axial pressure (such as 0.8 MPa) was applied to the transducer. However, for core samples containing oblique fractures, if a certain axial pressure was also applied, slippage and dislocation occurs at the fracture surfaces of the two core halves, resulting in the failure of the experiment. To avoid slippage and dislocation at the fracture surfaces under axial clamping pressure, it is necessary to apply a certain transversal clamping pressure on the core sample at the fracture position.

**Figure 6:**
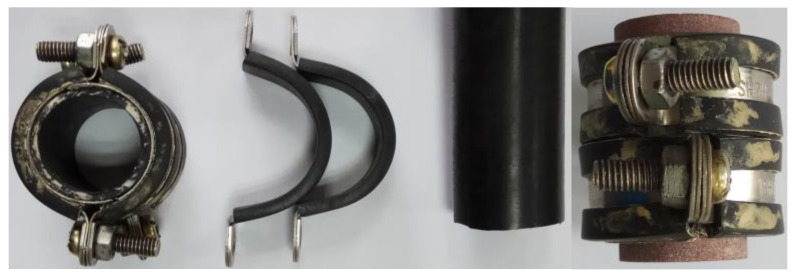
The transversal clamping device for core with oblique fracture.

**Figure 7:**
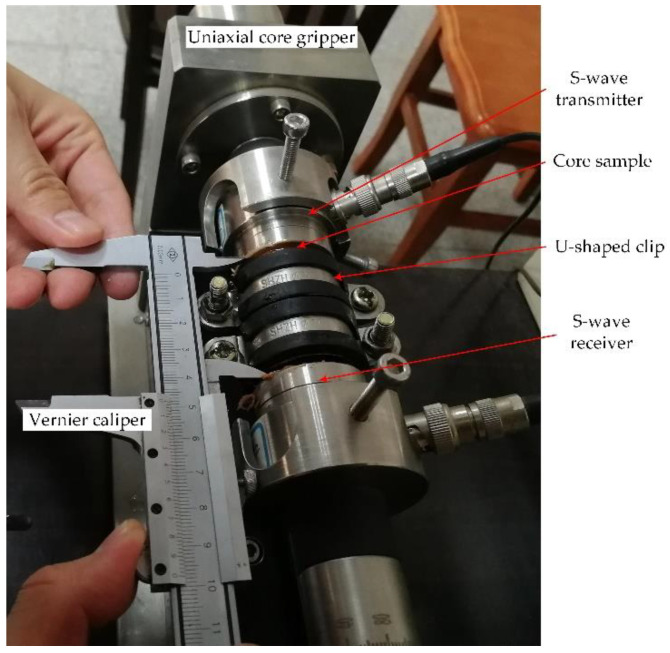
Photograph of the bidirectional clamped core with oblique fracture.

Therefore, U-shaped clips were selected to apply a certain transversal clamping pressure on the core. The transversal clamping device is shown in Figure 6. The clamping steps of the core with oblique fractures were as follows: first, an annular PET film gasket of appropriate specifications was placed between two core-cutting surfaces, and the two core halves were joined together along the fracture surfaces. Secondly, the core with an oblique fracture was placed in a rubber tube with an inner diameter approximately equal to the diameter of the core. Thirdly, two pairs of U-shaped clips with appropriate sizes were used to clamp the outside of the rubber tube, and the transversal clamping of the core with the oblique fracture was completed after tightening the screws of the clips. Finally, the transversally clamped core was clamped by the axial core gripper to achieve the bidirectional (transversal and axial) clamping (as detailed in Figure 7).

### 3.5. Experimental Measurement Method

**Figure 8:**
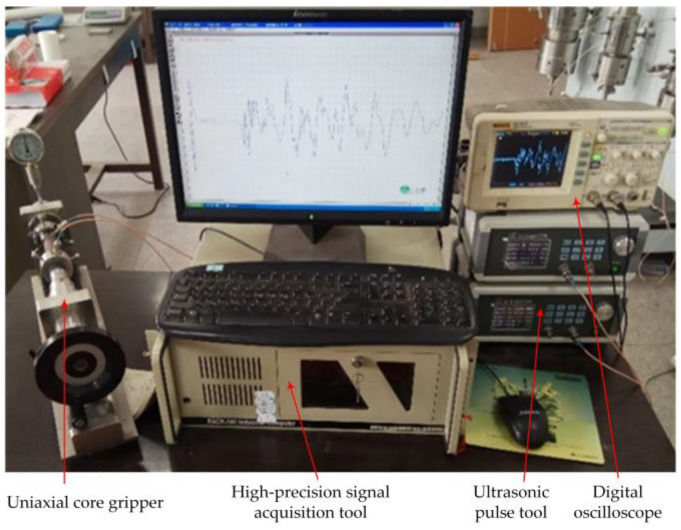
Photograph of the ultrasonic experimental measurement system, which mainly consists of two S-wave transducers, an ultrasonic pulse tool, a signal acquisition tool, a digital oscilloscope, and a core sample clamping device.

The experimental measurement can be conducted by the following method: first, the core containing a fracture with a 0° dip angle was selected and the fracture width was adjusted. Secondly, the core sample with a fracture was clamped bidirectionally and fixed in the experimental system. Thirdly, the measurement system parameters were set and the waveforms were measured. Finally, the core sample and fracture parameters were changed, and the above measurements were repeated. Our experiment was conducted under normal temperature conditions and the variation of the temperature was 18–28 °C. A photograph of the ultrasonic experimental measurement system is presented in Figure 8.

The complete experimental measurement steps were as follows: (1) the elliptic annular PET film gaskets of different specifications were prepared for cores containing fractures with different dip angles, and the gaskets with thicknesses of 25, 50, 75, 100, 150, 200, 300, 400, 500, and 1000 μm were needed according to the experimental scheme. To avoid the influence of the gasket on the experiment results, the gaskets with various thicknesses for the fracture of specified dip angle should have the same shape. (2) A core sample containing an oblique fracture was chosen, and a specification of PET film gasket was selected to place between the fracture faces with air filling within the annular PET film. Then, the core sample was clamped transversally. (3) The ultrasonic S-wave transmitting and receiving transducers were installed on the axial core gripper, and high viscosity butter was smeared uniformly on the two ends of the core sample, which was afterwards placed between the two transducers and clamped by the axial core gripper (as shown in Figure 7). The clamping pressure of the axial core gripper was adjusted to 0.8 MPa. It should be noted that the transmitting and receiving transducers must maintain the same polarization direction (see Figure 1). (4) The transducers were connected with the ultrasonic signal excitation and acquisition systems according to the schematic diagram of the measurement system presented in Figure 1. The ultrasonic pulse tool was used to apply an excitation signal with a frequency band of 20–150 kHz and amplitude of 50 V to the transmitting transducer. The ultrasonic signal passed through the core sample and was received by the receiving transducer and returned to the ultrasonic pulse tool, and the waveform signal was digitized and recorded by the high-precision signal acquisition tool (or oscilloscope). The measurement system parameters, such as excitation signal voltage, frequency band, synchronization signal level, gain, sampling interval (0.05 μs), and acquisition length (2048 samples), were set. The shear waveforms were measured and recorded, during which the length of the core with an oblique fracture was measured and recorded with a vernier caliper. It should be noted that the waveforms were recorded and stacked 32 times to improve the signal-to-noise ratio. (5) The fracture attribute parameters (fracture dip angle and width) were adjusted, while keeping all other experimental conditions unchanged. Steps (2)–(4) were repeated, while measuring and recording the shear waveforms of each set of cores under different fracture attribute parameters; (6) the experimental waveform data were processed and analyzed, after which the laws were summarized.

## 4. Experimental Data Processing and Analysis

**Figure 9:**
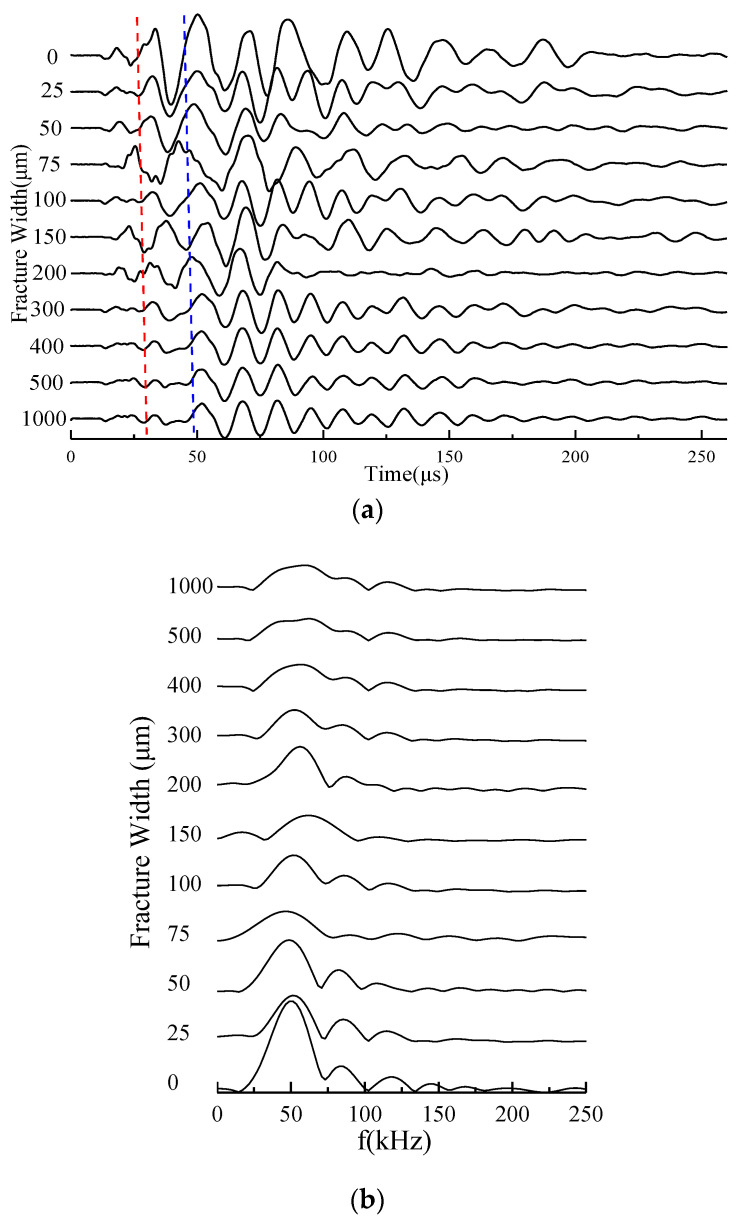
Experimental shear waveforms of core sample B1 at different fracture width conditions (**a**) and the corresponding S-wave spectrum (**b**). The red and blue dashed lines, respectively, illustrate the start and end positions of the target time window for S-wave-energy analysis in (**a**), and the positions of the red and blue dashed line indicators are determined by the S-wave arrivals and the length of the time window.

According to the above-mentioned methods, the shear waveform measurement experiments were performed on the 12 fractured core samples listed in Table 1 under different fracture width conditions. Figure 9 shows the shear waveforms obtained by the experimental measurement of the B1 core sample with a fracture dip angle of 0° under different fracture width conditions and the corresponding S-wave spectrum. The P- and S-wave arrivals can be estimated according to the P- and S-wave velocities of the core. According to the arrivals, we can find that the 1–2 cycles in the front of each full-waveform were the P-wave; and the P-wave was followed by the S-wave. It can be observed from Figure 9a that the S-wave amplitude (or energy) gradually decreases with the increase in the fracture width. Furthermore, Figure 9b illustrates that the frequency contents of the S-waveforms are similar.

**Figure 10:**
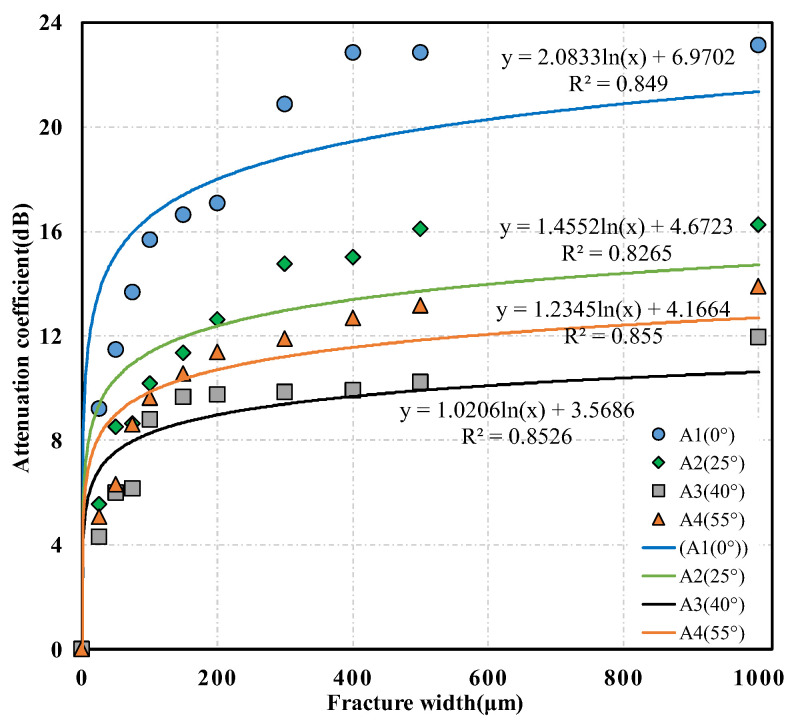
Relationship between shear wave attenuation coefficient and fracture width of the set of A cores containing fractures with different dip angles.

**Figure 11:**
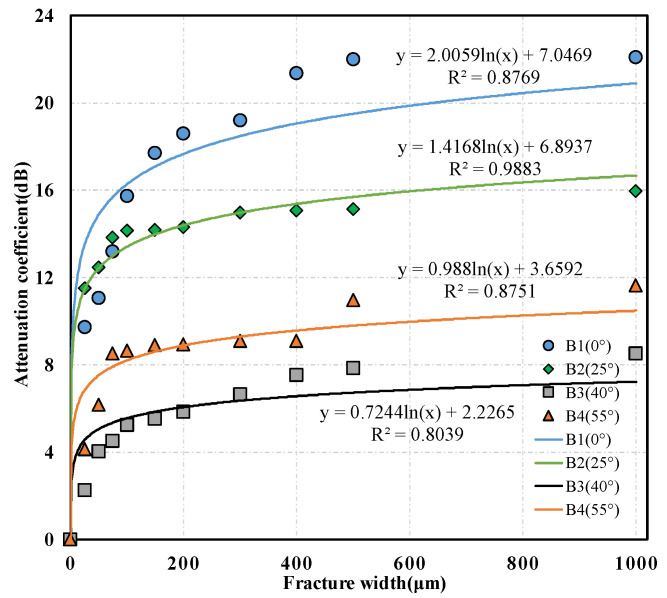
Relationship between shear wave attenuation coefficient and fracture width of the set of B cores containing fractures with different dip angles.

**Figure 12:**
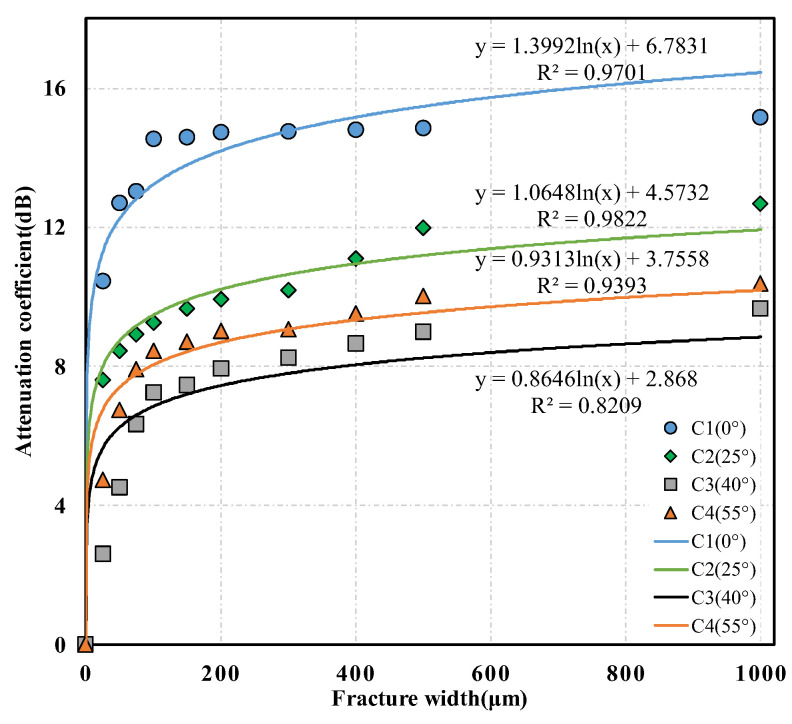
Relationship between shear wave attenuation coefficient and fracture width of the set of C cores containing fractures with different dip angles.

To quantitatively study the relationship between S-wave attribution parameters and the fracture width further, the S-wave energies in the target time windows of the waveforms measured in each core with an oblique fracture under different fracture width conditions were calculated. Then, the corresponding S-wave-energy attenuation coefficients were determined according to Formula (1). The 1–2 cycles in the front of the S-wave were generally selected as the target time windows, and the red and blue dashed lines, respectively, illustrated the start and end positions of the target time window in Figure 9a. Furthermore, the intersection diagrams of the S-wave attenuation coefficient and the fracture width were drawn under different fracture dip angle conditions and the quantitative relationship between the S-wave attenuation coefficient and the fracture width was analyzed. The relationships between the S-wave attenuation coefficient and the fracture width of the three sets of cores containing fractures with different dip angles are shown in Figure 10, Figure 11 and Figure 12, respectively, where the scattered points refer to the experimental results and the solid lines represent the corresponding logarithmic fitting results. According to the fitting curves, we find that the S-wave attenuation coefficient can be expressed as a function of the fracture width for all the cores with different porosity ranges and oblique fractures, which are detailed in the following Equations (2)–(4):

(2) $$\begin{array}{cc} \left(4\%\leq \Phi <5\%\right) & \alpha =\left\{ \begin{array}{l} \begin{array}{cc} 2.0833\ln(w)+6.9702 & {(0}^{\circ }) \end{array}\\ \begin{array}{cc} 1.4552\ln(w)+4.6723 & {(25}^{\circ }) \end{array}\\ \begin{array}{cc} 1.0206\ln(w)+3.5686 & {(40}^{\circ }) \end{array}\\ \begin{array}{cc} 1.2345\ln(w)+4.1664 & {(55}^{\circ }) \end{array} \end{array}\right. \end{array},$$

(3) $$\begin{array}{cc} \left(5\%\leq \Phi <6\%\right) & \alpha =\left\{ \begin{array}{l} \begin{array}{cc} 2.0059\ln(w)+7.0469 & {(0}^{\circ }) \end{array}\\ \begin{array}{cc} 1.4168\ln(w)+6.8937 & {(25}^{\circ }) \end{array}\\ \begin{array}{cc} 0.7244\ln(w)+2.2265 & {(40}^{\circ }) \end{array}\\ \begin{array}{cc} 0.9880\ln(w)+3.6592 & {(55}^{\circ }) \end{array} \end{array}\right. \end{array},$$

(4) $$\begin{array}{cc} \left(6\%\leq \Phi <8\%\right) & \alpha =\left\{ \begin{array}{l} \begin{array}{cc} 1.3992\ln(w)+6.7831 & {(0}^{\circ }) \end{array}\\ \begin{array}{cc} 1.0648\ln(w)+4.5732 & {(25}^{\circ }) \end{array}\\ \begin{array}{cc} 0.8646\ln(w)+2.8680 & {(40}^{\circ }) \end{array}\\ \begin{array}{cc} 0.9313\ln(w)+3.7558 & {(55}^{\circ }) \end{array} \end{array}\right. \end{array},$$

where α is the ultrasonic S-wave attenuation coefficient, dB; w is the fracture width, μm; and Φ is the core porosity, %.

It can be observed from Figure 10, Figure 11 and Figure 12 that the ultrasonic S-wave attenuation coefficient has a good correlation with the fracture attribute parameters. The S-wave attenuation coefficient rises logarithmically with the increase in the fracture width for the cores with different porosities and various fracture dip angles. The ultrasonic S-wave attenuation coefficient changes relatively faster with the fracture width when the fracture width is within 250 μm. This indicates that the S-wave attenuation is more sensitive to the change of microfracture width. Such a phenomenon can be explained by the acoustic attenuation theory. According to the acoustic attenuation theory, there are three main types: scattering attenuation, absorption attenuation, and diffusion attenuation [43]. When a micro-fracture appears, due to the apparent difference in acoustic impedance between the fracture filler and the matrix rock, an interface with an obvious acoustic impedance difference is formed at the fracture surface, which causes a significant decrease in the acoustic wave amplitude and a sharp increase in attenuation. In this situation, the attenuation change was mainly controlled by the scattering or reflection attenuation. As the fracture width increases, the scattering or reflection attenuation caused by the fracture interface gradually becomes stable. At this time, the attenuation change was mainly controlled by the absorption attenuation caused by the fracture filler, and the attenuation change caused by the absorption attenuation was slower than that caused by the scattering or reflection attenuation.

The comparison of the experimental results of cores with different porosities shows that the S-wave attenuations are different under the same fracture dip angle and width conditions. This indicates that the difference in core porosity affects the attenuation of the S-wave. The comparison of the experimental results of the three sets of cores containing fractures with different dip angles reveals that, with constant fracture width and similar core porosities, the S-wave attenuation caused by the 0° fracture is significantly greater than that caused by fractures with other dip angles. Sequentially, this is followed by the S-wave attenuations caused by 25° and 55° fractures. The S-wave attenuation caused by a 40° fracture is relatively minimal. This conclusion is consistent with the experimental results of Kpolov who is a scholar from the former Soviet Union [20]. This may be due to the fact that the polarization direction of the particles caused by the S-wave was approximately parallel to the fracture surface under the condition of a low dip angle fracture. When the S-wave propagates to the fracture and the fracture dip angle is low, the vibration path of the particles has a larger proportion in the fracture fluid, which may result in more energy loss and greater absorption attenuation.

The above quantitative results about the influence of fractures on shear wave attenuation are more accurate than the previous qualitative results [40,41], which can be used for guidance regarding further research on the nondestructive testing methods or evaluation methods of micro-fracture attribute parameters and effectiveness based on S-wave attenuation information. It should be noted that laboratory acoustic experiments are generally conducted under high-frequency conditions [26,29,30,31,34,35]. Therefore, in practical applications, if there is a significant difference (e.g., 10 times or more) between the field and our experimental acoustic frequency bands, the fracture scale should be adjusted in proportion, according to the acoustic similarity principle and the relationship between the acoustic frequencies of the application field and laboratory.

## 5. Conclusions

A physical experiment was conducted to quantitatively study the influence of micron-scale oblique fractures on S-wave attenuation for the problems of fracture parameters and effectiveness evaluation in tight sandstone reservoirs. The effects of the fracture dip angle and width on S-wave attenuation in three sets of tight sandstone cores with different porosity ranges were meticulously studied. According to the experimental measurements and data processing and analysis results of the above-mentioned 12 core samples under different fracture dip angles and fracture width conditions, the following conclusions were mainly drawn:

(1) This experimental scheme is feasible and can be applied to related investigation studies on the influence of fracture properties especially the micro-fracture properties and on ultrasonic wave propagation in a core with an oblique fracture.

(2) When the fracture dip angles are within 0°–60° and the fracture widths are within 0–1000 μm, the S-wave attenuation coefficient and fracture attribute parameters, especially the fracture width, have a good correlation, and the S-wave attenuation coefficients all rise logarithmically with the increase in the fracture width for cores with different porosities and different fracture dip angles, based on which the quantitative relationships between the S-wave attenuation coefficient and the fracture attribute parameters are established. Moreover, the S-wave attenuation coefficient changes relatively faster with the fracture width when the fracture width is within 250 μm. This indicates that the S-wave attenuation is more sensitive to the change in the microfracture width. It should be noted that the difference in core porosity affects the attenuation of the S-wave.

(3) When the fracture width is constant and the core porosities are similar, the S-wave attenuation caused by the 0° fracture is significantly greater than that caused by fractures with other dip angles. This is followed by the S-wave attenuations caused by 25° fracture and 55° fracture sequentially. The 40° fracture causes the relatively smallest S-wave attenuation.

The results of this study can be used to guide further research on the nondestructive testing methods or evaluation methods for microfracture attribute parameters and effectiveness based on S-wave attenuation information, so as to make up for the shortcomings of the image logging fracture evaluation method and further improve the fracture evaluation method system. The study results can also provide guidance for the research of amplitude compensation methods for sonic signal transmission in fractured media. It should be noted that the influence of the flat fracture width and dip angle on S-wave attenuation was studied in this paper, principally through rock physical experiments. The influences of other factors, such as sonic frequency, fracture roughness, fracture filling condition, and fracture width greater than 1000 μm on acoustic wave attenuation and the corresponding correction schemes, still need further research. Furthermore, the evaluation method for reservoir fractures based on borehole shear wave data and experimental results also needs further research, especially the calculation method for borehole shear wave attenuation caused by a fracture and the relationship between borehole S-wave attenuation and experimental S-wave attenuation.

## Figures and Tables

**Table 1:** Physical parameters of the experimental cores.

Set	Core No.	Fracture Dip	Core Diameter	Core Length	Porosity
		°	mm	mm	%
A	A1	0	24.80	47.08	4.88
	A2	25	25.30	48.42	4.97
	A3	40	24.60	48.44	4.92
	A4	55	25.30	49.22	4.97
B	B1	0	25.34	46.02	5.77
	B2	25	25.28	48.72	5.48
	B3	40	25.30	49.28	5.61
	B4	55	25.30	49.94	5.70
C	C1	0	25.30	44.96	7.09
	C2	25	25.00	48.10	7.10
	C3	40	25.30	49.26	6.59
	C4	55	24.74	49.82	7.07

## Data Availability

The data presented in this study are available on request from the corresponding author.
